# Large-scale ^13^C-flux analysis reveals mechanistic principles of metabolic network robustness to null mutations in yeast

**DOI:** 10.1186/gb-2005-6-6-r49

**Published:** 2005-05-17

**Authors:** Lars M Blank, Lars Kuepfer, Uwe Sauer

**Affiliations:** 1 Institute of Biotechnology, ETH Zürich, 8093 Zürich, Switzerland

## Abstract

**Background:**

Quantification of intracellular metabolite fluxes by ^13^C-tracer experiments is maturing into a routine higher-throughput analysis. The question now arises as to which mutants should be analyzed. Here we identify key experiments in a systems biology approach with a genome-scale model of *Saccharomyces cerevisiae *metabolism, thereby reducing the workload for experimental network analyses and functional genomics.

**Results:**

Genome-scale ^13^C flux analysis revealed that about half of the 745 biochemical reactions were active during growth on glucose, but that alternative pathways exist for only 51 gene-encoded reactions with significant flux. These flexible reactions identified *in silico *are key targets for experimental flux analysis, and we present the first large-scale metabolic flux data for yeast, covering half of these mutants during growth on glucose. The metabolic lesions were often counteracted by flux rerouting, but knockout of cofactor-dependent reactions, as in the *adh1*, *ald6*, *cox5A*, *fum1*, *mdh1*, *pda1*, and *zwf1 *mutations, caused flux responses in more distant parts of the network. By integrating computational analyses, flux data, and physiological phenotypes of all mutants in active reactions, we quantified the relative importance of 'genetic buffering' through alternative pathways and network redundancy through duplicate genes for genetic robustness of the network.

**Conclusions:**

The apparent dispensability of knockout mutants with metabolic function is explained by gene inactivity under a particular condition in about half of the cases. For the remaining 207 viable mutants of active reactions, network redundancy through duplicate genes was the major (75%) and alternative pathways the minor (25%) molecular mechanism of genetic network robustness in *S. cerevisiae*.

## Abstract 2

Genome-scale 13^C^-flux analysis in *Saccharomyces cerevisiae* revealed that the apparent dispensability of knockout mutants with metabolic function can be explained by gene inactivity under a particular condition, by network redundancy through duplicated genes or by alternative pathways.

## Background

The availability of annotated genomes and accumulated biochemical evidence for individual enzymes triggered the reconstruction of stoichiometric reaction models for network-based pathway analysis [1,2]. For many microbes, such network models are available at the genome scale, providing a largely comprehensive metabolic skeleton by interconnecting all known reactions in a given organism [3,4]. Thus, network properties such as optimal performance, flexibility to cope with ever-changing environmental conditions, and enzyme dispensability (also referred to as robustness or genetic robustness [5,6]) become mathematically tractable. These computational advances are matched with post-genomic advances in experimental methods that assess the cell's molecular make-up at the level of mRNA, protein, or metabolite concentrations. As the functional complement to these compositional data, quantification of intracellular *in vivo *reaction rates or molecular fluxes has been a focal point of method development in the realm of metabolism [7-9]. Recent progress in increasing the throughput of stable-isotope-based flux analyses [8,10,11] has allowed the quantification of flux responses to more than just a few intuitively chosen genetic or environmental perturbations [12-14]. Now that flux quantification in hundreds of null mutants under a particular condition is feasible in principle, the question arises of which mutants should be analyzed.

As perhaps the most widely used model eukaryote, the yeast *Saccharomyces cerevisiae *features a metabolic network of about 1,200 reactions that represent about 750 biochemically distinct reactions [3,15]. Is it necessary to quantify flux responses to null mutations in all reactions for a comprehensive view of the metabolic capabilities under a given condition? To address this question, we used a recently modified version (iLL672; L Kuepfer, U Sauer and LM Blank, unpublished work) of the original iFF708 genome-scale model published by Förster *et al. *[3]. On the basis of this model, we estimated the genome-scale flux distribution in wild-type *S. cerevisiae *from ^13^C-tracer experiments, to identify the 339 biochemical reactions that were active during growth on glucose. Yeast metabolism has the potential flexibility to use alternative pathways for 105 of these active reactions. For a major fraction of the potentially flexible reactions that catalyze significant flux, we then constructed prototrophic knockout mutants to elucidate whether or not the alternative pathway was used upon experimental knockout; that is, whether it contributes to the genetic robustness of the network [5,6]. For the purpose of this work, robustness is defined as the ability to proliferate on glucose as the sole carbon source upon knockout of a single gene with metabolic function.

## Results

### Identification of flexible reactions in yeast metabolism

To identify all potentially flexible reactions in yeast glucose metabolism that were active under a given condition, we used the recently reconciled metabolic network model iLL672 with 1,038 reactions (encoded by 672 genes) that represent 745 biochemically distinct reactions (L Kuepfer, U Sauer and LM Blank, unpublished work), which was based on the genome-scale *S. cerevisiae *model iFF708 [3]. The main modifications to the original model include elimination of dead-end reactions and a new formulation of cell growth. It should be noted that none of the results below critically depended on the network model, but the reconciliation of iLL672 enabled a more accurate discrimination between lethal and viable reactions than iFF708, as was validated by large-scale growth experiments (L Kuepfer, U Sauer and LM Blank, unpublished work).

**Figure 1:**
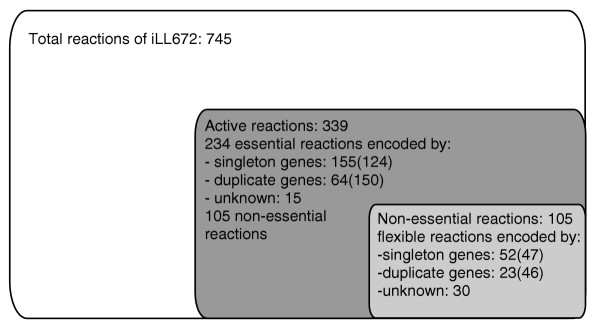
Genome-wide proportion of active, essential and flexible metabolic reactions during growth of *S. cerevisiae *(iLL672) on glucose. Flexible reactions are defined as having a non-zero flux but are not essential for growth. The number of genes that encode biochemical reactions is given in parentheses.

First, we identified all reactions active in wild-type glucose metabolism by genome-scale flux analysis. For this purpose, we determined the wild-type flux distribution in central metabolism from a stable isotope batch experiment with 20% [U-^13^C] and 80% unlabeled glucose. This flux solution was then mapped to the genome scale by using minimization of the Euclidean norm of fluxes as the objective function. In total, 339 of the 745 biochemical reactions were active during growth on glucose alone (Figure 1 and Additional data file 1), which agrees qualitatively with the estimate of Papp *et al. *[16]. Most active reactions (234) were essential: 155 are encoded by singleton genes, 64 by two or more duplicate genes and 15 by yet unknown genes (Figure 1; Additional data file 1). In the entire network, only the remaining 105 reactions (30 encoded by yet unknown genes) were active and potentially flexible in the sense that they may be bypassed via alternative pathways (Figure 1). As fluxes in the peripheral reactions were typically below 0.1% of the glucose uptake rate (see Additional data file 1), we focused on the 51 gene-encoded flexible reactions that catalyzed a flux of at least 0.1%. These 51 reactions were encoded by 75 genes (43 duplicates, 23 singletons and 9 multiprotein complexes).

### Physiological fitness of mutants deleted in flexible reactions

**Figure 2:**
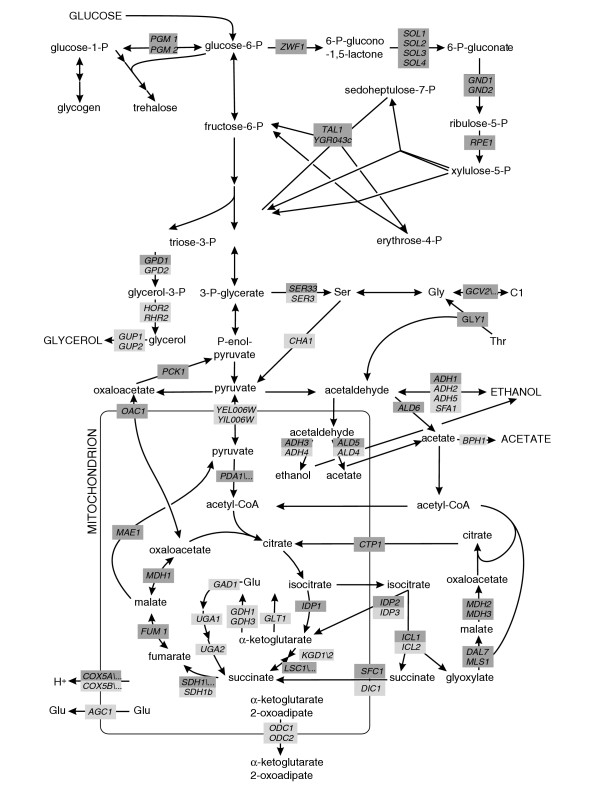
Central carbon metabolism of *S. cerevisiae *during aerobic growth on glucose. Gene names in boxes are given for reactions that were identified as flexible by flux balance analysis. Dark gray boxes indicate mutants, for which the carbon flux distribution was determined by ^13^C-tracer experiments. Dots indicate that the gene is part of a protein complex. Arrowheads indicate reaction reversibility. Extracellular substrates and products are capitalized. C1, one-carbon unit from C_1 _metabolism.

In 38 of these genes, which encoded 28 of the 51 potentially flexible and highly active reactions, we constructed prototrophic deletion mutants by homologous recombination [17] in the physiological model strain CEN.PK [18] (Figure 2). The prototrophic background was chosen to minimize potential problems of amino-acid supplementation for quantitative analysis [19]. These 38 experimental knockouts were in the pentose phosphate (PP) pathway, tricarboxylic acid (TCA) cycle, glyoxylate cycle, polysaccharide synthesis, mitochondrial transporters, and by-product formation (Figure 2, Table 1). Genetically, the knockouts encompass 14 singleton and 24 duplicate genes, including six gene families of which all members were deleted.

With the exception of *gnd1*, all 38 mutants grew with glucose as the sole carbon source. The lethal phenotype of the *gnd1 *mutant is consistent with a previous report [20] and is similar to the *gndA *mutant in *Bacillus subtilis *[21]. As in *B. subtilis*, we could select *gnd1 *suppressor mutants on glucose (data not shown). To assess the quantitative contribution of each gene to the organism's fitness, we determined maximum specific growth rates in minimal and complex medium using a well-aerated microtiter plate system [22]. Mutant fitness was then expressed as the normalized growth rate, relative to the growth rate of the reference strain (Table 1). In contrast to the previously reported competitive fitness [20,23,24], the fitness determined here is a quantitative physiological value.

In complex YPD medium, physiological fitness in the 38 viable haploid mutants was generally in qualitative agreement with the competitive fitness [20]. Quantitatively, however, our data seem to allow a better discrimination (Table 1), and significant differences between physiological and competitive fitness were seen in the *adh1*, *fum1*, and *gpd1 *mutants. Only threemutants - *adh1*, *fum1*, and *gly1 *- exhibited a fitness defect of 20% or greater (Table 2). *gly1 *lacks threonine aldolase, which catalyzes cleavage of threonine to glycine [25], hence its phenotype remains unexplained because glycine was present in the YPD medium.

In general, growth on the single substrate reduced the metabolic flexibility, as a much greater proportion of mutants exhibited significant fitness defects (Table 2). Major fitness defects were prominent in mutants of the PP pathway (*gnd1*, *rpe1*, *sol3*, and *zwf1*), which indicates an increased demand of NADPH for biosynthesis. Fitness of the *fum1 *mutant was clearly lower than that of other TCA-cycle mutants, for which duplicate genes exist. The strong phenotype of the *fum1 *mutant was somewhat unexpected because the flux through the TCA cycle is generally low or absent in glucose batch cultures of *S. cerevisiae *[13,14,26,27].

### Intracellular carbon flux redistribution in response to gene deletions

**Figure 3:**
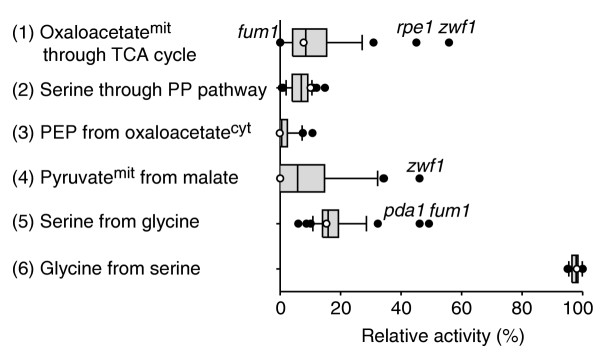
The distribution of six independently determined metabolic flux ratios in 37 deletion mutants during growth on glucose. In each case, the median of the distribution is indicated by a vertical line, the 25th percentile by the grey box and the 90th percentile by the horizontal line. Data points outside the 90th percentile are indicated by dots. The reference strain is indicated by the open circle.

While physiological data quantify the fitness defect, they cannot differentiate between intracellular mechanisms that bring about robustness to the deletion. To identify how carbon flux was redistributed around a metabolic lesion, we used metabolic flux analysis based on ^13^C-glucose experiments [8,9]. In contrast to *in vitro *enzyme activities and expression data, ^13^C-flux analysis provides direct evidence for such *in vivo *flux rerouting or its absence. The flux protocol consists of two distinct steps: first, analytical identification of seven independent metabolic flux ratios with probabilistic equations from the ^13^C distribution in proteinogenic amino acids [12,28,29]; and second, estimation of absolute fluxes (*in vivo *reaction rates) from physiological data and the flux ratios as constraints [10,30]. The relative distribution of intracellular fluxes was rather invariant in the 37 mutants, with the fraction of mitochondrial oxaloacetate derived through the TCA cycle flux and the fraction of mitochondrial pyruvate originating from malate as prominent exceptions (Figure 3).

**Figure 4:**
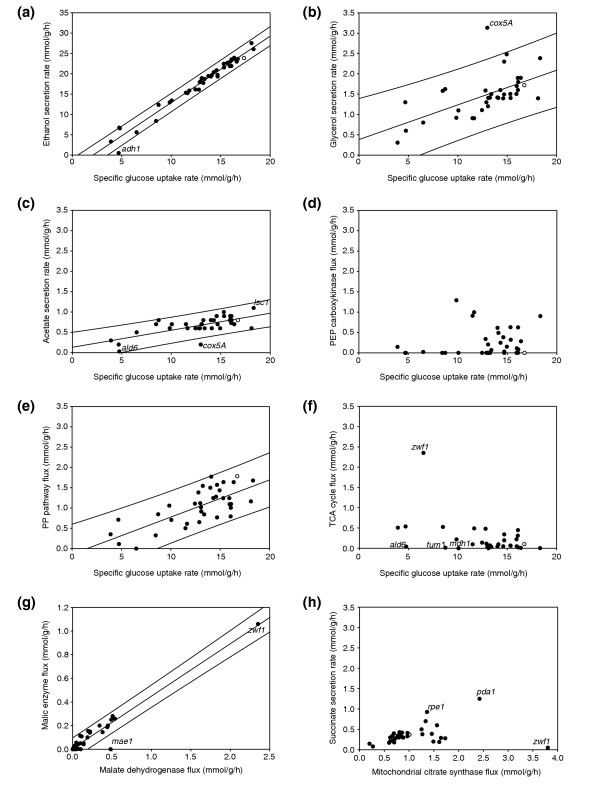
Absolute metabolic fluxes in the 37 flexible mutants as a function of glucose uptake rate or selected intracellular fluxes. **(a-f) **Glucose uptake rate; **(g,h) **selected intracellular fluxes. The linear regression of the distribution and the 99% prediction interval are indicated by the solid and dashed lines, respectively. Mutants with significant changes in the carbon-flux distribution are indicated. The reference strain is indicated by an open circle. Extreme flux patterns were verified in 30-ml shake flask cultures (data not shown).

From the experimentally determined uptake/production rates and the flux ratios as constraints (Additional data file 3), absolute intracellular fluxes were calculated using a compartmentalized stoichiometric model that consists of 35 reactions and 30 metabolites (Additional data file 2). This flux model comprised mostly the reactions of central carbon metabolism that were most relevant to the genetic changes introduced. It should be noted that the deleted reactions, with the exception of pyruvate dehydrogenase (*PDA1*), were not omitted from the network model; thus the calculated absence of flux through a given reaction was independently verified from the ^13^C-labeling data. In contrast to the relative distribution of intracellular fluxes, absolute reaction rates varied significantly in the mutants. With the exception of the flux through the TCA cycle (Figure 4f) and the gluconeogenic PEP carboxykinase (Figure 4d), all other fluxes generally increased with increasing glucose uptake rates (Figure 4). Eleven of the 37 mutants, however, exhibited specific flux responses that deviated from this general trend (Table 2, Figure 4).

### Specific flux responses in singleton and duplicate gene knockouts

**Figure 5:**
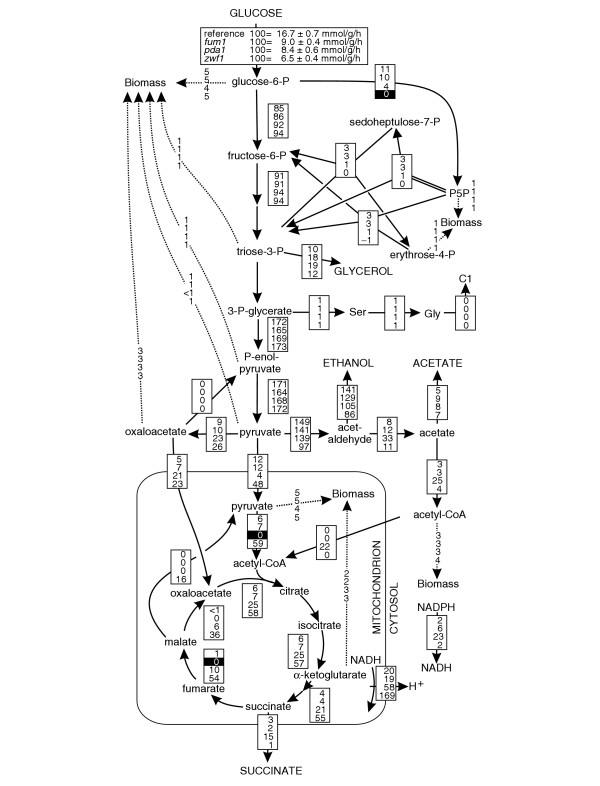
Relative distributions of absolute carbon fluxes in the *S. cerevisiae *reference strain (Ref) and the singleton gene mutants *fum1*, *pda1 *and *zwf*. All fluxes are normalized to the specific glucose uptake rate, which is shown in the top inset, and are given in the same order in each box. Reactions encoded by deleted genes are shown on a black background, but were not removed from the flux model (except for *PDA1*). The NADPH balance that is based on the quantified fluxes and the known cofactor specificities is given as a synthetic transhydrogenase flux. In general, the 95% confidence intervals were between 5 and 10% for the major fluxes. Larger confidence intervals were estimated for reactions with low flux such as malic enzyme and PEP carboxykinase. Flux distributions were verified in 30-ml shake flask cultures (data not shown). C1, one-carbon unit from C_1 _metabolism; P5P, pentose 5-phosphates.

Specific flux responses were more prominent among the singleton mutants (Table 2, Figure 4). Although the TCA cycle flux through the NAD^+^-dependent fumarase reaction from fumarate to malate was already very low in the reference strain (Figures 3, 4f), the *fum1 *mutant exhibited a pronounced phenotype with altered redox metabolism and significant glycerol production (Figure 5). Inactivation of the mitochondrial pyruvate dehydrogenase complex in the *pda1 *mutant was bypassed by the import of cytosolic acetyl-CoA into the mitochondria. Inactivation of the oxidative PP pathway branch in the *zwf1 *mutant was compensated by a reversed flux in the non-oxidative PP pathway to provide the biomass precursors pentose 5-phosphate and erythrose 4-phosphate (Figure 5). Because the primary role of the PP pathway on glucose is generation of NADPH, NADP^+^-dependent mitochondrial malic enzyme flux was significantly increased in the *zwf1 *mutant. This NADPH compensation by malic enzyme was also suggested recently from co-feeding experiments [31].

**Figure 6:**
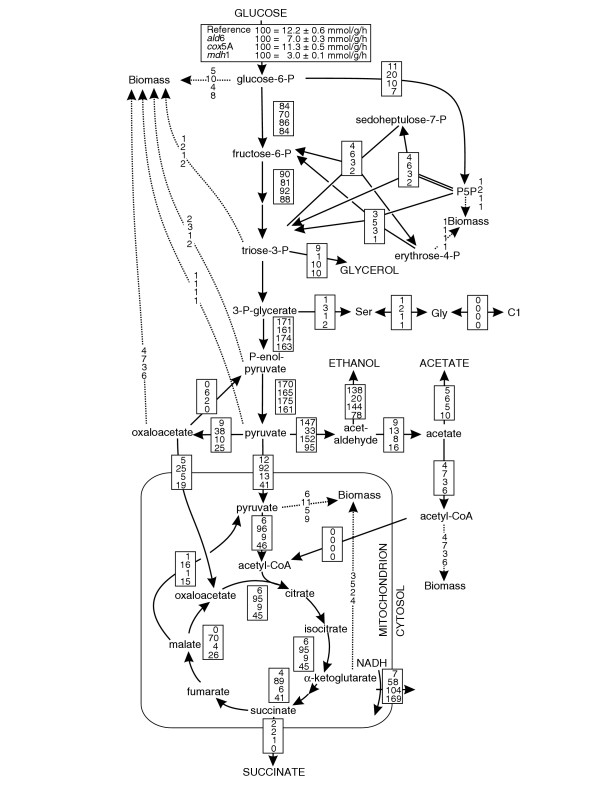
Relative distributions of absolute carbon fluxes in the *S. cerevisiae *reference strain and the duplicate gene mutants *ald6*, *cox5A *and *mdh1*. All fluxes are normalized to the specific glucose uptake rate, which is shown in the top inset, and are given in the same order in each box. Reactions encoded by deleted genes are shown on a black background, but were not removed from the flux model. The NADPH balance that is based on the fluxes and the known cofactor specificities is given as a synthetic transhydrogenase. In general, the 95% confidence intervals were between 5 and 10% for the major fluxes. Larger confidence intervals were estimated for reactions with low flux such as malic enzyme and PEP carboxykinase. Flux distributions were verified in 30-ml shake flask cultures (data not shown). C1, one-carbon unit from C_1 _metabolism; P5P, pentose 5-phosphates.

In contrast to singletons, deletion of flexible duplicate genes could be compensated by either alternative pathways or isoenzymes. In most cases, however, the isoenzymes were used because no flux alteration was detected, with the a *dh1*, *ald6*, *cox5A*, and *mdh1 *mutants as exceptions (Table 2). Deletion of the major acetate-producing acetaldehyde dehydrogenase, the cytoplasmic *ALD6 *[32], significantly reduced acetate formation. The primary effect of the deletion was the strongly reduced glucose-uptake rate (Figure 4). Although a major source of NADPH was inactivated in this mutant [33], the PP pathway flux was not increased, but was even lower than in the reference strain (Figure 6). This indicates that the strongly decreased fitness of the *ald6 *mutant (Table 1) could result from NADPH starvation - that is, a suboptimal rate of NADP^+ ^reduction. Consistent with this, we estimated that the NADPH requirement exceeded the combined NADPH formation from the oxidative PP pathway and malic enzyme by 70%, indicating that an as-yet-unidentified reaction(s) substitutes for the remaining NADPH production. Candidates are the mitochondrial acetaldehyde dehydrogenase Ald4p [34], which can use either NAD^+ ^or NADP^+ ^as redox cofactors or the mitochondrial NADH kinase Pos5p [35]. Deletion of the cytochrome *c *oxidase subunit Va *COX5A *in the mitochondrial respiratory chain increased glycerol production, which serves as means to reoxidize NADH (Figures 4b, 6). Because this mutant lacks functional mitochondria [36], glycerol production was driven by the limited NADH reoxidation through residual NADH oxidase activity in the electron-transport chain. Thus, robustness was brought about by using an alternative NADH sink. Considering that the flux through the mitochondrial malate dehydrogenase Mdh1 was already very low in the reference strain, the fitness defect of the *mdh1 *was surprising. Akin to the *fum1 *and *ald6 *mutants, the significantly reduced fitness of *mdh1 *may be explained by the imbalance between the TCA cycle and glucose catabolism (Figure 4f). Generally, the TCA cycle flux increases with decreasing glucose uptake rates [29], but remains non-proportionally low (absent) in the *fum1*, *ald6*, and *mdh1 *mutants (Figure 4f). The cytosolic and peroxisomal duplicate genes *MDH2 *and *MDH3*, respectively, did not compensate for the mitochondrial lesion, which is consistent with the observed lethal phenotype of the *mdh1 *mutant when grown on acetate [37].

### Genetic network robustness

The above flux results reveal that knockouts of flexible reactions are bypassed through alternative pathways in about one third of the cases and through isoenzymes in the other two thirds. Does this reflect the relative contribution of alternative pathways and duplicate genes to genetic network robustness? [5] To address this question quantitatively for glucose metabolism, we grew the 196 duplicate (encoding 87 reactions) and 171 singleton (encoding 207 reactions) knockout mutants of all 294 gene-encoded active reactions on glucose plates.

**Figure 7:**
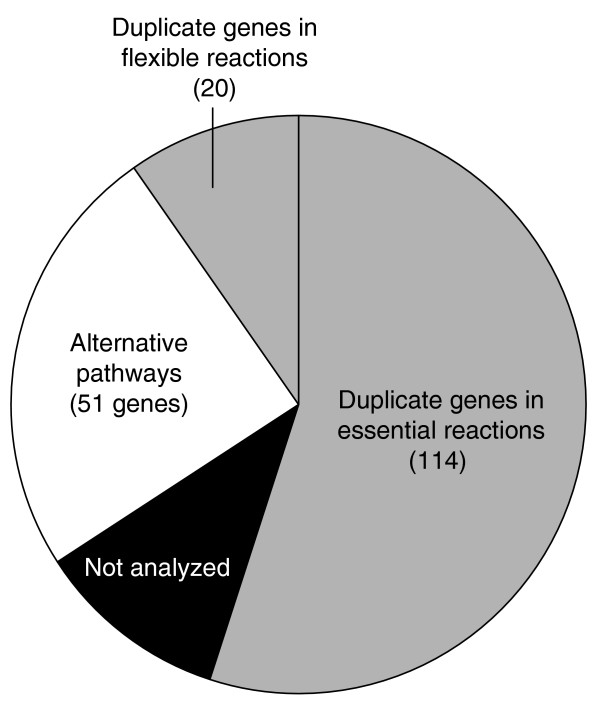
The mechanistic basis of gene dispensability in all active reactions during glucose metabolism of *S. cerevisiae*. The mechanism was mostly identified from the phenotype on glucose plates. For 10 of the alternative pathways and for 20 duplicates encoding flexible reactions, the results were confirmed by ^13^C-flux analysis. For 22 duplicate genes the data are not sufficient to distinguish between both mechanisms and they are labeled as not analyzed.

In the 47 viable singleton knockouts, flux rerouting through an alternative pathway ensures survival, which was directly verified by flux data in 10 cases (Figure 4, Table 3 and Additional data file 3). Of the 196 experimental duplicate mutants, 180 grew on sole glucose, while 16 of the mutations were lethal. As these 16 duplicates obviously did not contribute to genetic robustness, their entire families (36 genes) were subtracted from the 150 duplicate-encoded essential reactions (Figure 1). For the remaining 114 duplicate genes we have strong evidence for network redundancy as the underlying mechanism of robustness, because they encode essential reactions (as determined *in silico*) and each of the experimental knockouts was viable (Figure 7). For the 46 duplicate genes that encode flexible reactions (Figure 1), both compensation by duplicates and/or alternative pathways might ensure proliferation. Where available, these mutants were classified according to their flux distribution; that is, of the 24 experimental duplicate mutants analyzed, four used alternative pathways and 20 an isoenzyme (Figure 4, Table 3 and Additional data file 3). In total we analyzed all 367 experimental mutants that encode the 294 active reactions of glucose metabolism, 140 of which were lethal and 227 viable. For the vast majority of the viable mutants, we identified the molecular mechanism that brought robustness to the knockout about: about 25% were alternative pathways and 75% duplicate genes (Figure 7).

## Discussion

Using an integrated computational and experimental approach, we show here that metabolic flexibility to knockout mutations is restricted to a relatively small set of biochemical reactions. About a third of all active reactions under the particular condition investigated may be bypassed by alternative pathways, of which about 30% support only negligible fluxes. The occurrence of flexible reactions might be even lower in prokaryotes, because several alternative pathways involved inter-compartmental transport. In general, the number of flexible reactions will differ substantially between species, with free-living yeast and fungi at the upper end of the scale, and intracellular pathogens with highly reduced genomes at the lower end.

Using flux balance (FBA) [1,2], elementary flux mode [38,39], or similar analyses [40], all *in silico *flexible reactions can be precisely identified. Hence, experimental analysis of intracellular flux responses to metabolic gene deletions can be limited to these potentially flexible mutants, rather than having to analyze the entire mutant collection. Using the systems biological approach described here, the true *in vivo *capability of metabolic network operation can be mapped with a reasonable workload. As the knowledge base on intracellular flux responses increases, a handful of flux experiments in computationally identified mutants will probably suffice to identify the *in vivo *network capability under a given condition. At the next level, such flux analyses will also include mutants affected in regulatory genes that modulate the network composition. Although not covered in the stoichiometric models employed for flux-balance analysis, several recently discussed computational approaches [39,41,42] may aid in identifying the most relevant regulatory mutants for *in vivo *flux quantification.

Consistent with the notion that metabolic networks undergo minimal flux redistributions with respect to the metabolic state of the parent [40], deletion of flexible singleton genes was mostly counteracted by local flux rerouting, for example, the *lsc1*,*mae1*, and *oac1 *mutants (see Additional data file 3). Deletions in redox cofactor-dependent singleton or duplicate reactions such as those mediated by *adh1*, *ald6*, *cox5A*, *pda1*, and *zwf1*, however, affected flux alterations in more distant reactions. While the relative flux distribution (in % values) was perturbed only very little in these mutants, the absolute magnitude of fluxes (*in vivo *reaction velocities) varied dramatically. In particular, knockout of *fum1*, whose encoded protein catalyzes only a rather small flux, led to an unexpectedly strong phenotype with about a 50% reduction in glucose-uptake rate. Although unexpected, this finding was qualitatively consistent with results from a recent genetic footprinting study [43], which also showed a significant fitness defect in this mutant. It was speculated that intramitochondrial shortage of amino acids such as aspartate and glutamate causes a lack of respiratory chain components, which leads to a petite-like phenotype [44]. Another key mutant was *pda1*, whose knockout caused a substantial import of acetyl-CoA into the mitochondria; the mechanism for this remains elusive because the carnitine auxotrophic CEN.PK strain does not use the carnitine shuttle [45]. As a consequence, a twofold overproduction of NADPH was estimated, which suggests that the NAD^+^-dependent acetaldehyde dehydrogenases instead of the NADP^+^-dependent *ALD6 *were active to balance NADPH formation/consumption. Consistent with this, the flux through the NADPH-producing PP pathway was significantly lower in this mutant. The strongly altered redox metabolism in *pda1 *is further evidenced by the substantial secretion of glycerol and succinate (Figure 5).

The metabolic flexibility to cope with metabolic lesions is generally known as genetic robustness [5], a concept that is used to explain the seemingly surprising number of phenotypically silent deletion mutations: only about 1,100 knockouts of the 5,700 genes are lethal in haploid *S. cerevisiae *[23,46]. The causes and evolution of gene dispensability have been investigated in several theoretical analyses of pre-existing data, but the issue remains controversial [5,6,16,47-49]. For metabolic networks, our flux data differentiate between the relative contributions of three mechanisms to the apparent genetic robustness: inactive, and thus dispensable, genes; 'genetic buffering' through alternative reactions; and functional complementation from duplicate genes ('redundancy').

## Conclusions

In qualitative agreement with a recent estimate [16], genome-scale flux analysis revealed that about half of the available reactions (45% of the known metabolic genes) were not required for growth on glucose (Figure 1). Hence, deletion of these genes would not affect the growth phenotype on this substrate, making inactive reactions the primary reason for the apparent dispensability of genes with metabolic function. It should be noted that this apparent gene dispensability is a somewhat artificial classification that does not contribute to genetic robustness because most of these genes encode metabolic functions that are only relevant under conditions different from the one tested. The most important mechanism of true genetic robustness in yeast glucose metabolism was duplicate genes (Figure 7), the majority of which encoded essential reactions with no alternative pathway. Alternative pathways, contributed about 25% to genetic robustness by carbon flux rerouting. This leaves redundancy as the major, and modularity the minor, cause [50] of metabolic network robustness to single-gene deletions during growth on glucose.

## Materials and methods

### Yeast strains

All prototrophic *S. cerevisiae *deletion mutants were constructed in the haploid, CEN.PK113-7D (*Mat***a ***MAL2-8*^*c *^*SUC2*) background with the homolog flanking region approach [17] (Table 1). Briefly, genomic DNA was isolated from the corresponding amino-acid auxotrophic mutants [23]. The *kanMX4 *cassettes of each mutant were amplified by PCR with primers located about 500 bp upstream and downstream of the deleted genes. The PCR reaction mixture was directly used for transformation and integrants were selected on YPD plates with 300 *μ*g/ml geneticin. Correct cassette insertion was confirmed by overlapping PCR using either primer *Kan*B (5'-CTGCAGCGAGGAGCCGTAAT-3') or *Kan*C (5'-TGATTTTGATGACGAGCGTAA-3') primers in combination with one gene-specific primer. The reference strain was CEN.PK 113-7D with a deletion of the switching endonuclease, which was shown to be phenotypically neutral in chemostat competition experiments [51] and is commonly used as reference [52,53].

### Media and growth conditions

The composition of the yeast minimal medium (MM) was [54]: 5 g (NH_4_)_2_SO_4_, 3 g KH_2_PO_4_, 0.5 g MgSO_4_·7H_2_O, 4.5 mg ZnSO_4_·7H_2_O, 0.3 mg CoCl_2_·6H_2_O, 1.0 mg MnCl_2_·4H_2_O, 0.3 mg CuSO_4_·5H_2_O, 4.5 mg CaCl_2_·2H_2_O, 3.0 mg FeSO_4_·7H_2_O, 0.4 mg NaMoO_4_·2H_2_O, 1.0 mg H_3_BO_3_, 0.1 mg KI, 15 mg EDTA, 0.05 mg biotin, 1.0 mg calcium pantothenate, 1.0 mg nicotinic acid, 25 mg inositol, 1.0 mg pyridoxine, 0.2 mg *p*-aminobenzoic acid, and 1.0 mg thiamine. The medium was buffered at pH 5.0 with 100 mM KH-phthalate to reduce pH changes throughout the growth experiments to 0.05. Filter-sterilized glucose and geneticin (300 μg/ml) were added freshly to the media. Batch growth experiments (1.2 ml) were carried out in deep-well plates (System Duetz, Kühner AG, Switzerland) using an orbital shaker with 5 cm amplitude at 300 rpm to allow optimal mixing [22].

Qualitative testing of mutant growth on glucose was done on agar plates. For this purpose, we used the haploid yeast mutant library in the BY4741 strain (*MAT***a ***his3Δ1 leu2Δ0 met15Δ0 ura3Δ0*) [23]. The composition of the yeast minimal medium for the plate growth assay was as described above [54] with the exception of 20 g/l agar for solidification. Glucose was added to a final concentration of 20 g/l. Strain auxotrophies were complemented with 20 mg/l histidine, uracil, methionine, lysine and 60 mg/l leucine. The plates were incubated at 30°C for 3 days before scoring of the growth phenotype and further incubated for 1 week to score slow-growth phenotype mutants.

### Analytical procedures and ^13^C-labeling experiments

Cell growth was monitored by following optical density changes at a wavelength of 600nm (OD_600_). Aliquots for extracellular metabolite analysis were centrifuged at 14,000 rpm in an Eppendorf tabletop centrifuge to remove cells. Glucose, acetate, ethanol and glycerol concentrations in the supernatant were determined with commercial enzymatic kits (Scil Diagnostics, Germany). Organic acids were quantified by high-pressure liquid chromatography (HPLC) using a Supelcogel C8 (4.6 by 250 mm) ion-exclusion column. The column was eluted at 30°C with 2% sulfuric acid at a flow rate of 0.3 ml/min. The organic acids were detected using a PerkinElmer UV detector (Series 2000) at a wavelength of 210 nm. The physiological parameters maximum specific growth rate, biomass yield on glucose, and specific glucose consumption rate were calculated during the exponential growth phase.

All labeling experiments were carried out in batch cultures assuming pseudosteady-state conditions during the exponential growth phase [12,55]. ^13^C-labeling of proteinogenic amino acids was achieved either by growth on 5 g/l glucose as a mixture of 80% (w/w) unlabeled and 20% (w/w) uniformly labelled [U-^13^C]glucose (^13^C > 99%; Martek Biosciences, Columbia, MD) or 100% [1-^13^C]glucose (> 99%; Omicron Biochemicals, South Bend, IN). Cells from overnight cultures were harvested by centrifugation and washed using sugar-free MM to remove residual unlabeled carbon sources. Cultures were routinely inoculated to an maximum OD_600 _of 0.03 and harvested by centrifugation at an OD_600 _≤ 1. Residual medium was removed by washing the pellet with water. Cell protein was hydrolyzed for 24 h at 105°C in 6 M HCL and dried in a heating block at 85°C for 6 h. The free amino acids were derivatized at 85°C for 1 h using 15 μl dimethylformamide and 15 μl *N*-(*tert*-butyldimethylsilyl)-*N*-methyl-trifluoroacetamide [10]. Gas chromatography-mass spectrometry (GC-MS) analysis was carried out as reported [12] using a series 8000 GC in combination with an MD800 mass spectrometer (Fisons Instruments, Beverly, MA).

### Metabolic flux ratio analysis

The recorded MS spectra include the distribution of mass isotopomers in 1-5 fragments of alanine, aspartate, glutamate, glycine, isoleucine, leucine, phenylalanine, proline, serine, threonine, tyrosine, and valine. For each amino-acid fragment *α*, a mass isotopomer distribution vector (MDV) was assigned:



with *m*_0 _being the fractional abundance of the lowest mass and *m*_*i*>0 _the abundances of molecules with higher masses. The MDV_*α *_values were corrected for naturally occurring stable isotopes [12] to obtain the exclusive mass isotopomer distributions of the carbon skeletons. The corrected MDV_*α *_were used to calculate the amino acids (MDV_AA_) and metabolites (MDV_M_) mass distribution vectors. Ratios of converging intracellular fluxes to a given metabolite were calculated from the MDV_M _as described previously [12,29].

In addition, the relative contribution of the PP pathway was quantified from [1-^13^C]glucose experiments by tracking the positional ^13^C-labeling [10,56]. The expected labeling pattern of triose phosphates or serine, which is derived exclusively through glycolysis, is 50% ^13^C-label in the C_1 _positions. Hence, the fraction of serine derived through the pentose phosphate (PP) pathway can be derived according to Equation 2 [12]:



where GLU3_unlabeled _is an unlabeled three-carbon fragment from a source molecule of glucose. The remaining fraction of serine must then be derived through glycolysis. This flux ratio was not corrected for the potential withdrawal of ^13^C-label in dihydroxyacetone-phosphate-based biomass synthesis (such as phospholipids) and glycerol formation [21], because the influence was negligible under the condition used. The largest effect was found in the mutant with the highest specific glycerol formation rate (*cox5A*), where the estimated relative flux through the PP pathway would decrease from 12 ± 1% to 9 ± 1%.

### ^13^C-constrained flux analysis

Absolute values of intracellular fluxes were calculated with a flux model comprising all the major pathways of yeast central carbon metabolism (Additional data file 2). Deleted reactions were not omitted from the mutant models; thus the mutations were independently verified from the ^13^C data. The stoichiometric matrix of 34 linear equations and 30 metabolites has an infinite condition number [57]; it is thus underdetermined, and has a solution space with an infinite number of different flux vectors that fulfill the constraints from determined uptake and production rates. To uniquely solve the system for fluxes (*ν*), a set of linearly independent equations that quantify flux ratios (FlRs) were used to obtain eight constraints on the relative flux distribution from METAFoR analysis (see Additional data file 2).

The fraction of cytosolic oxaloacetate originating from cytosolic pyruvate is given by:



The fraction of mitochondrial oxaloacetate derived through anaplerosis is given by:



The fraction of PEP originating from cytosolic oxaloacetate is given by:



The fraction of serine derived through glycolysis is given by:



The upper and lower bounds for mitochondrial pyruvate derived through the malic enzyme (from mitochondrial malate) are given by:



The contribution of glycine to serine biosynthesis is given by:



and, finally, the contribution of serine to glycine biosynthesis is given by:



The stoichiometric matrix including Equations 3-10 has a condition number of 31, implying that the model is numerically robust [57]. Error minimization was carried out as described by Fischer *et al*. [10]. Balanced NADPH production and consumption were not added as additional constraints. In general, NADPH production was constrained by Equations 3 and 7/8, which estimate the relative use of the PP pathway and malic enzyme, respectively. As an additional source of NADPH, the flux through the NADPH-dependent acetaldehyde dehydrogenase [33] was estimated from the acetate production rate and the biomass requirement for cytosolic acetyl-CoA. Deviation of the NADPH production estimated in this way from the consumption for biosynthesis was generally below ± 20%, suggesting that the model assumptions and the experimental data are highly consistent. All extreme flux patterns were independently verified in 30-ml cultures (data not shown).

### Genome-scale flux analysis

We used the experimentally determined *in vivo *flux data (*ν*^*exp*^) to constrain the purely stoichiometric solution space of model iLL672 to obtain an experimentally validated genome-scale wild-type flux solution *ν*^*WT*^. For glucose minimal medium, we constrained the model iLL672 with 30 fluxes that were derived from ^13^C-labeling experiments [8]. In particular, we used ^13^C-constrained flux analysis [58] for GC-MS-detected mass isotope distributions in proteinogenic amino acids from a 20% [U-^13^C] glucose experiment and a compartmentalized yeast model [29]. These experimental data were to be kept within an accuracy *δ *of ± 10% when mapping the determined central metabolic fluxes to the genome-scale reference flux solution. To overcome mathematical artifacts such as futile cycling (that is, a closed loop of fluxes that bring no net change), the original linear programming problem was modified. A minimization of the Euclidean norm of fluxes was chosen as the objective function such that (s.t.) the mass balance equations hold:



with *j *as the set of experimentally determined fluxes. Reactions were categorized as flexible when fulfilling the following criteria: the reactions carried a non-zero flux; and the reaction was not essential for growth.

### *In silico *phenotyping of duplicate gene families

Phenotype predictions of deletion mutants were analyzed computationally with FBA [3,59]. Assuming steady-state growth, mass balances were put up for each intracellular metabolite *M*_*i *_(1 × *n*) that have to be fulfilled, when multiplied with the overall flux vector *ν *(*n *× 1):

*M*_*i*_·*ν *= 0.     (12)

The entity of all *m *metabolite mass balances yields the stoichiometric matrix *S *(*m *× *n*), where:

S·*ν *= 0.     (13)

To pick one solution out of the overall solution space formed by the stoichiometric constraints, FBA generally assumes maximization of biomass growth *μ *as the global cellular goal [3,59]. Thus, the search for a single flux distribution *ν *results in a linear programming (LP) problem:



where *i *= 1,..,*M *and *ν*_*lb*,*i *_and *ν*_*ub*,*i *_correspond to upper and lower bounds of a specific reaction *i*. Gene knockout mutants can be simulated easily *in silico *by setting the deleted reactions to zero. All LPproblems were solved using the open-source GNU linear programming kit [60].

## Additional data files

The following additional data are available with the online version of this paper. The classification of reactions according to Figure 1 is presented in Additional data file 1. The flux analysis model is defined in Additional data file 2. The physiological data, flux ratios and the calculated flux distributions are presented in Additional data file 3.

## Supplementary Material

Additional File 1 Classification of reactions according to Figure 1. Classification of reactions according to Figure 1 Click here for file

Additional File 2 The flux analysis model. The flux analysis model Click here for file

Additional File 3 Physiological data, flux ratios and the calculated flux distributions. Physiological data, flux ratios and the calculated flux distributions Click here for file

## Figures and Tables

**Table 1:** Fitness of mutants with deletions in flexible central metabolic reactions

	Physiological fitness*		Competitive fitness^†^		Physiological fitness		Competitive fitness
					
Mutants	MM	YPD	YPD	Mutants	MM	YPD	YPD
Reference strain	1	1	1				
*adh1*/YOL086C	0.47	0.57	0.79	*mdh2*/YOL126C	0.89	0.98	1.01
*adh3*/YMR083W	0.92	0.87	0.98	*mdh*3/YDL078C	1.00	0.96	1.01
*ald5*/YER073W	1.02	0.94	1	*mls1*/YNL117W	1	0.98	1
*ald6*/YPL061W	0.34	0.87	0.9	*oac1*/YKL120W	0.71	0.94	1.01
*cox5A*/YNL052W	0.63	0.91	1	*pck1*/YKR097W	1	0.96	1
*ctp1*/YBR291C	0.91	1	0.97	*pda1*/YER178W	0.41	0.98	1
*dal7*/YIR031C	0.94	0.85	1	*pgm1*/YKL127W	0.82	0.94	1
*fum1*/YPL262W	0.52	0.62	0.93	*pgm2*/YMR105C	0.90	1	1
*gnd1*/YHR183W	0	0.87	1.01	*rpe1*/YJL121C	0.33	0.94	0.88
*gnd2*/YGR256W	0.83	0.98	1	*sdh1*/YKL148C	0.72	0.94	1
*gcv2*/YMR189W	0.92	0.94	1	*ser33*/YIL074C	0.92	0.94	1.01
*gly1*/YEL046C	0.79	0.74	0.87	*sfc1*/YJR095W	0.84	0.96	1.01
*gpd1*/YDL022W	1	0.98	0.84	*sol1*/YNR034W	0.91	1	1.02
*icl1*/YER065C	1	1	1	*sol2*/YCRX13W	0.99	0.98	1
*idp1*/YDL066W	0.92	0.94	1.03	*sol3*/ YHR163W	0.71	0.94	1
*idp2*/YLR174W	0.86	0.96	0.95	*sol4*/ YGR248W	0.95	0.91	1.01
*lsc1*/YOR142W	1.05	0.93	1	*tal1*/ YLR354C	0.89	0.94	1
*mae1*/YKL029C	1.01	0.96	1	YGR043C	0.92	0.87	1.02
*mdh1*/YKL085W	0.72	0.91	1	*zwf1*/YNL241C	0.38	0.96	ND

*Physiological fitness is defined as the maximal specific growth rate of a mutant normalized to the reference strain CEN.PK 113-7D *ho****::****kanMX4*. The average from triplicate experiments is shown. The standard deviation was generally below 0.05. ^†^From Steinmetz *et al*. [20]. ND, not detected.

**Table 2:** Overview of mutants with a fitness defect of at least 20% or altered flux distribution

Mutants	Fitness defect in YPD		Fitness defect in MM		Altered intracellular flux distribution*	
		
Total number of mutants	3 of 38		12 (+1)^† ^of 38		11 of 38	
						
Singleton genes	*fum1*	*gly1*	*fum1*	*pda1*	*fum1*	*pda1*
			*gly1*	*rpe1*	*lsc1*	*rpe1*
			*oac1*	*zwf1*	*mae1*	*zwf1*
					*oac1*	
						
Duplicate genes	*adh1*		*adh1*	*sdh1*	*adh1*	*cox5A*
			*ald6*	*sol3*	*ald6*	*mdh1*
			*cox5A*	(*gnd1*)		
			*mdh1*			

*See Figures 5 and 6. ^†^Lethal mutations are given in parentheses.

**Table 3:** Overview of mechanisms of metabolic flexibility that confer robustness to central metabolic deletions

Duplicate gene*	Duplicate gene and alternative pathway^†^	Alternative pathway^‡^	None
		
*ADH3*, *ALD5*, *DAL7*, *GPD1*,*ICL1, IDP1*, *IDP2*, *MDH2, MDH3, MLS1, PGM1*, *PGM2*, *SDH1*, *SER33*,*SOL1*, *SOL2*, *SOL3*, *SOL4*, *TAL1*, *YGR043c*	*ADH1*, *ALD6*, *COX5A*,*MDH1*	*FUM1*, *GLY1*, *LSC1*, *MAE1*, *MDH1, OAC1*, *PCK1*, *PDA1*, *RPE1*, *ZWF1*	*CTP1*, *GCV2*, *GND1*^§^, *GND2*, *SFC1*

*Wild-type-like flux distribution. ^†^Altered flux distribution, but some residual flux through the reaction was observed. ^‡^Altered flux distribution, but no residual flux through the reaction was observed. ^§ ^Lethal, probably because of a non-stoichiometric effect.
