# Supplementary material for: Large-scale 13C-flux analysis reveals mechanistic principles of metabolic network robustness to null mutations in yeast
Source: Genome Biol. 2005 May 17;6(6):R49. doi: 10.1186/gb-2005-6-6-r49 (PMC1175969; doi:10.1186/gb-2005-6-6-r49)
Supplement: Additional File 2 — The flux analysis model. The flux analysis model [file gb-2005-6-6-r49-S2.doc]

**Additional data file 2.** The metabolic flux analysis model used in this study.

|  | Metabolic network: |  |  |
| --- | --- | --- | --- |
| v1 | Glucose + ATP -> glucose-6-P | v18 | succinate <-> fumarate + NADH |
| v2 | glucose-6-P <-> fructose-6-P | v19 | malate <-> *^mit^*oxaloacetate + NADH |
| v3 | glucose-6-P -> P5P + 2 NADPH + CO_2_ | v20 | fumarate <-> malate |
| v4 | fructose-6-P + ATP -> 2 triose-3-P | v21 | malate -> *^mit^*pyruvate + CO_2_ + NADPH |
| v5 | 2 P5P <-> sedoheptulose-7-P + triose-3-P | v22 | *^cyt^*oxaloacetate + ATP -> PEP + CO_2_ |
| v6 | P5P + erythrose-4-P <-> fructose-6-P + triose-3-P | v23 | *^cyt^*pyruvate + CO_2_ + ATP-> *^cyt^*oxaloacetate |
| v7 | sedoheptulose-7-P + triose-3-P <-> erythrose-4-P + fructose-6-P | v24 | acetate + 2 ATP <-> *^cyt^*acetyl-CoA |
| v8 | triose-3-P -> serine + NADH | v25 | acetaldehyde <-> acetate + NADPH |
| v9 | serine + NADH -> glycine + C1 | v26 | acetaldehyde + NADH <-> CO_2_ + ethanol |
| v10 | glycine + C1 -> serine + NADH | v27 | triose-3-P + NADH <-> glycerol |
| v11 | C1 + CO_2_ + NADH <-> glycine | v28 | *^cyt^*oxaloacetate -> *^mit^*oxaloacetate |
| v12 | triose-3-P <-> PEP + ATP + NADH | v29 | *^mit^*oxaloacetate -> *^cyt^*oxaloacetate |
| v13 | PEP -> *^cyt^*pyruvate + ATP | v30 | *^cyt^*acetyl-CoA -> *^mit^*acetyl-CoA |
| v14 | *^mit^*pyruvate -> *^mit^*acetyl-CoA + CO_2_+ NADH | v31 | *^cyt^*pyruvate -> *^mit^*pyruvate |
| v15 | *^mit^*oxaloacetate + *^mit^*acetyl-CoA -> citrate | v32 | O_2_ + 2 NADH -> 2 P/O x ATP |
| v16 | citrate -> α-ketoglutarate + CO_2_+ NADH | v33 | *^cyt^*pyruvate -> acetaldehyde + CO_2_ |
| v17 | α-ketoglutarate -> succinate + CO_2_ + 0.5ATP + NADH | v34 | Biomass |
|  | Flux ratios: |  |  |
| FlR1 | *^cyt^*oxaloacetate from *^cyt^*pyruvate | FlR5 | *^mit^*pyruvate from *^mit^*oxaloacetate (upper bound) |
| FlR2 | *^mit^*oxaloacetate from anaplerosis | FlR6 | *^cyt^*pyruvate from *^mit^*oxaloacetate (lower bound) |
| FlR3 | PEP from *^cyt^*oxaloacetate | FlR7 | serine from glycine |
| FlR4 | serine through glycolysis | FlR8 | glycine from serine |

Abbreviations: PEP, phosphoenolpyruvate; P5P, pentose-5-phosphates; *^cyt^*, cytosolic; *^mit^*, mitochondrial.
